# Genetically Divergent Highly Pathogenic Avian Influenza A(H5N8) Viruses in Wild Birds, Eastern China

**DOI:** 10.3201/eid2711.204893

**Published:** 2021-11

**Authors:** Guimei He, Le Ming, Xiang Li, Yuhe Song, Ling Tang, Min Ma, Jie Cui, Tianhou Wang

**Affiliations:** East China Normal University, Shanghai, China (G. He, L. Ming, L. Tang, M. Ma, T. Wang);; CAS Key Laboratory of Molecular Virology & Immunology, Institut Pasteur, Center for Biosafety Mega-Science, Chinese Academy of Sciences, Shanghai (X. Li, Y. Song, J. Cui)

**Keywords:** H5N8, avian influenza virus, wild birds, China, highly pathogenic avian influenza, viruses, respiratory infections, influenza

## Abstract

In late 2020, we detected 32 highly pathogenic avian influenza A(H5N8) viruses in migratory ducks in Shanghai, China. Phylogenetic analysis of 5 representative isolates identified 2 sublineages of clade 2.3.4.4b. Each sublineage formed separate clusters with isolates from East Asia and Europe.

Highly pathogenic avian influenza (HPAI) A(H5Nx) clade 2.3.4.4 viruses, which originated from the HPAI A(H5N1) clade 2.3.4 of the A/Goose/Guangdong/1/96-lineage in China, have spread globally, causing severe disease in poultry and wild birds (*1*–*4*). According to the World Health Organization, clade 2.3.4.4 viruses have evolved into 8 subclades, designated as clades 2.3.4.4a–h (https://www.who.int/influenza/vaccines/virus/202002_zoonotic_vaccinevirusupdate.pdf). In 2013, a novel reassortant A(H5N8) clade 2.3.4.4b virus was isolated from domestic ducks in eastern China (*2*); this virus was later detected in Korea and Japan (*3*). Since 2014, clade 2.3.4.4b viruses have spread to Europe and Africa along the migratory flyways of birds (*4*,*5*). These introductions caused large HPAI outbreaks in wild and domestic birds in Europe during the winter of 2016–17 (*6*). At the same time, wild birds carried clade 2.3.4.4c viruses to North America (*4*).

In early 2020, outbreaks of clade 2.3.4.4b viruses mainly occurred in Europe (*7*). Beginning in July 2020, several outbreaks of H5N8 viruses in poultry and wild birds were reported in Eurasia, including Kazakhstan, Russia, Poland, England, Netherlands, Korea, and Japan (*7*–*10*); outbreaks were not reported in China until October 2020, when clade 2.3.4.4b viruses related to those circulating in Eurasia were detected in 2 dead swans in Mongolia (*11*). Because eastern China is a major bird migration destination, migratory birds might carry HPAI viruses to this region. We detected 32 H5N8 viruses of 2 genetically distinct lineages in wild birds in eastern China.

## The Study

On October 31, 2020, we began annual surveillance for avian influenza viruses (AIVs) in migratory birds. As of December 2, 2020, we had collected 612 cloacal and tracheal swab samples from migratory ducks in the Jiuduansha wetland (31°06′–31°14′N, 121°46′–122°15′E). This wetland is located at the Yangtze River Estuary and is a major stopover site for migratory birds traveling along the East Asian–Australasian flyway. The birds showed no signs of illness. Reverse transcription PCR detected 32 H5N8 viruses by described procedures (*12*). We determined the prevalence of H5N8 viruses to be 5.2%. These H5N8-positive bird species comprised the common teal (*Anas crecca*), spot-billed duck (*Anas poecilorhyncha*), northern pintail (*Anas acuta*), falcated teal (*Anas falcata*), and mallard (*Anas platyrhynchos*). We determined the sequences of the hemagglutinin (HA) and neuraminidase (NA) gene segments of these isolates. We found that 31 isolates had nearly identical HA and NA segments, sharing 99.7%–100% nucleotide sequence identity. We then determined the entire genomic sequences of 5 representative isolates from 5 different host species. We designated these 5 isolates as A/common teal/Shanghai/JDS20103116/2020-H5N8 (GenBank accession nos. MW269587–94), A/northern pintail/Shanghai/JDS20843/2020-H5N8 (GenBank accession nos. MW362179–86), A/falcated teal/Shanghai/JDS20857/2020-H5N8 (GenBank accession nos. MW362170–7), A/spot-billed duck/Shanghai/JDS20867/2020-H5N8 (GenBank accession nos. MW362161–8), and A/mallard/ Shanghai/JDS20876/2020-H5N8 (GenBank accession nos. MW357308–15).

Whole-genome sequencing of these 5 H5N8 viruses revealed that isolate JDS20103116-H5N8 shared a relatively low nucleotide sequence identity (92.4%–97.8%) with the other 4 isolates, indicating that these viruses are genetically divergent. BLAST analysis (https://blast.ncbi.nlm.nih.gov/Blast.cgi) showed that these 5 H5N8 isolates shared the highest sequence identity (99.3%–100.0%) with H5N8 viruses isolated in late 2020 from poultry and wild birds in South Korea, Japan, and Europe (including Russia, Netherlands, and England) (Table). To further characterize these 5 isolates, we constructed phylogenetic trees by comparing the sequences of all 8 genomic segments with those in the GISAID database (https://www.gisaid.org) using IQ-TREE (*13*). We used the general time reversible (GTR) plus F plus G4 model for the HA and polymerase basic 2 protein segments, the transversion e plus G4 model for the matrix protein segment, the K3Pu plus F plus G4 model for the nonstructural protein and NA segments, the transversion plus F plus G4 model for the polymerase acidic protein segment, the GTR plus F plus invariant sites plus G4 model for the polymerase basic 1 protein segment, and the transition 2 plus F plus invariant sites plus G4 model for the nucleoprotein segment. We set parameters to –m (model selection), MFP (model find program), –B (ultrafast bootstrap value), 1,000 bootstraps, –T (threads for used for tree building), and AUTO (automatically selected number of threads). 

**Table:** Nucleotide sequence identity of 5 representative avian influenza A(H5N8) isolates from 5 different host species, Shanghai, China, 2020

Isolates	Gene segment	Homologous strains*	GISAID accession no.	Identity, %
JDS20103116	Polymerase basic 2 protein	A/duck/Korea/H439/2020 (A/H5N8)	EPI1845982	99.9
	Polymerase basic 1 protein	A/duck/Korea/H439/2020 (H5N8)	EPI1845983	99.9
	Polymerase acidic protein	A/northern pintail/Hokkaido/M13/2020(H5N8)	EPI1818401	99.7
	Hemagglutinin	A/duck/Korea/H439/2020 (A/H5N8)	EPI1845985	99.8
	Nucleoprotein	A/duck/Korea/H439/2020 (A/H5N8)	EPI1845978	99.3
	Neuraminidase	A/ duck/Korea/H439/2020 (A/H5N8)	EPI1845984	99.9
	Matrix protein	A/chicken/Kagawa/11C/2020(H5N8)	EPI1815028	99.8
	Nonstructural protein	A/chicken/Kagawa/11C/2020(H5N8)	EPI1815027	99.4
JDS20843, JDS20843, JDS20867, JDS20876	Polymerase basic 2 protein	A/wild duck/Korea/H331/2020 (H5N8)	EPI1846695	99.6
		A/spot-billed duck/Korea/WA1000/2020 (H5N8)	EPI1846695	99.6
	Polymerase basic 1 protein	A/quail/Korea/H440/2020 (H5N8)	EPI1846512	99.7
		A/chicken/Korea/H440/2020 (H5N8)	EPI1845991	99.7
	Polymerase acidic protein	A/chicken/Omsk/0119/2020 (H5N8)	EPI1813381	99.9
		A/domestic duck/kazakhstan/1–274–20-B/2020 (H5N8)	EPI1811610	99.9
	Hemagglutinin	A/chicken/Korea/H544/2020 (H5N8)	EPI1850622	100.0
		A/chicken/Korea/H001/2021 (H5N8)	EPI1846522	100.0
	Nucleoprotein	A/mallard/Kagoshima/ KU-d89/2021 (H5N8)	EPI1846675	99.9
		A/spot-billed duck/Korea/Wa1000/2020 (H5N8)	EPI1846697	99.9
	Neuraminidase	A/wild duck/Korea/H331/2020 (H5N8)	EPI1850682	99.9
		A/chicken/Korea/H002/2021 (H5N8)	EPI1846529	99.9
	Matrix protein	A/wild bird/Korea/H496–3/2020 (H5N8)	EPI1857465	100.0
		A/chicken/Korea/H544/2020 (H5N8)	EPI1850662	100.0
	Nonstructural protein	A/Greylag_goose/England/033100/2020 (A/H5N8)	EPI1837929	99.8
		A/turkey/Omsk/0003/2020 (H5N8)	EPI1846695	99.8

*List comprises the most or the 2 most homologous viruses available in GISAID (https://www.gisaid.org) for each gene segment.

Results showed that the isolates belonged to clade 2.3.4.4b and formed 2 distinct genetic sublineages (Figure). The isolate JDS20103116-H5N8 clustered with the isolates found in East Asia (including South Korea and Japan) in late 2020, as well as the isolates found in Europe in late 2019 and early 2020 (*14*). The other 4 H5N8 isolates clustered with the viruses found in poultry and wild birds in Eurasia (including South Korea, Japan, China, and Europe) in late 2020. The cluster showed high bootstrap support; we proposed the clade to be a novel genotype of the 2.3.4.4b clade (Figure). The topologic structure of trees based on the other gene segments were identical to that of the tree based on the HA gene segment (Appendix Figure).

**Figure:**
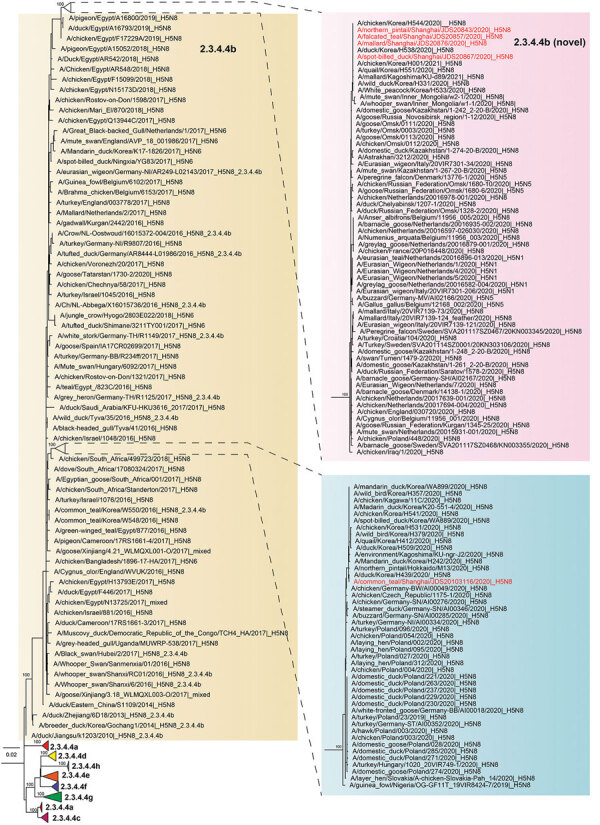
Phylogenetic tree of highly pathogenic avian influenza A(H5N8) viruses isolated in Shanghai, China during 2020 (red text) and reference sequences from GISAID (https://www.gisaid.org). Tree was based on hemagglutinin gene segment; it was constructed using IQ-TREE (*13*) with 1,000 bootstrap replicates. Yellow shading indicates subclade 2.3.4.4b strains. Pink and blue shading indicate 2 sublineages of subclade 2.3.4.4b. Numbers to the left of node indicate bootstrap values. Scale bars indicate average number of nucleotide substitutions per site.

The novel genotype of the 2.3.4.4b clade also was closely related to viruses detected in poultry in Iraq in May 2020 and in Egypt in 2019, suggesting that these viruses might be the source of the novel genotype. After the outbreaks in Iraq, clade 2.3.4.4b viruses were detected in backyard poultry in Russia in late July 2020 and in wild birds in Russia and Kazakhstan in September 2020. In October 2020, those viruses were also prevalent among birds traveling along various migratory flyways of Europe and Asia (*7**–**10*). We speculate that these viruses circulated among domestic birds in Egypt and then among migratory birds in Russia before emerging in Eurasia in late 2020. Because of the lack of surveillance data at breeding sites in 2019 and early 2020, the transmission routes of these viruses remain unclear. In February 2021, an avian influenza H5N8 infection was reported in a person in Russia. The causative virus, designated A/Astrakhan/3212/2020H5N8, belonged to the 2.3.4.4b clade. These observations suggest that the H5N8 viruses in this novel genotype of 2.3.4.4b clade could infect a wide range of hosts and might spread globally, as did previous H5N8 outbreaks that spread from Asia to Europe and North America in 2014 (*15*).

Molecular analysis of HA cleavage sites demonstrated that the 5 H5N8 isolates contain multiple basic amino acids, PLREKRRKR/GL, which are characteristic of HPAI viruses. The HA1 receptor-binding sites of all 5 H5N8 isolates have amino acid residues Q226 and G228 (H3 numbering), indicative of an avian-like (a2, 3-SA) receptor-binding preference. We documented 2 new amino acid substitutions, T140A and N236D (H3 numbering), in the HA protein of the novel genotype of the 2.3.4.4b clade. The significance of these 2 new mutations remains undetermined. We did not find the E627K and D701N residues in the polymerase basic 2 protein, suggesting that the viruses have not adapted to mammal hosts.

## Conclusions

During our annual surveillance, we detected 32 H5N8 HPAI viruses from migratory ducks without signs of illness in Shanghai, China. Results of phylogenetic analyses of 5 representative isolates showed that they belonged to 2 sublineages of H5N8 viruses circulating in this region. Some isolates clustered with a novel genotype of 2.3.4.4b clade that was identified in Europe and East Asia in late 2020. The detection of these H5N8 AIVs in asymptomatic migratory birds support the hypothesis that free-living wild birds play a crucial role in the dissemination of these viruses. More active surveillance is needed to detect new AIVs, especially in the breeding grounds and migratory sites of various birds. Because of their high genetic diversity, new AIVs might pose a substantial threat to global health.

Appendix Additional information on genetically divergent highly pathogenic avian influenza A(H5N8) viruses in wild birds, Eastern China.
