# Supplementary material for: Genetically Divergent Highly Pathogenic Avian Influenza A(H5N8) Viruses in Wild Birds, Eastern China
Source: Emerg Infect Dis. 2021 Nov;27(11):2940–3. doi: 10.3201/eid2711.204893 (PMC8544973; doi:10.3201/eid2711.204893)
Supplement: Appendix — Additional information on genetically divergent highly pathogenic avian influenza A(H5N8) viruses in wild birds, Eastern China. [file 20-4893-Techapp-s1.pdf]

Article DOI: https://doi.org/10.3201/eid2711.204893

Genetically Divergent Highly Pathogenic Avian Influenza A(H5N8) Viruses in Wild Birds, Eastern China

Appendix.

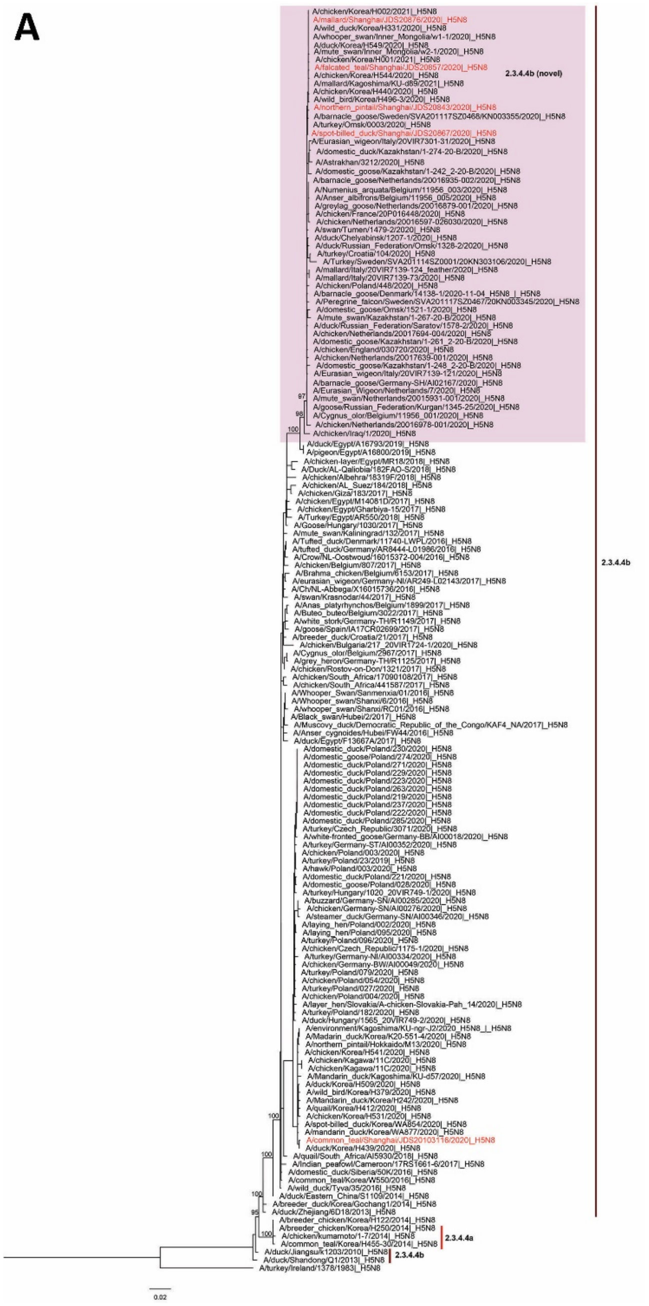

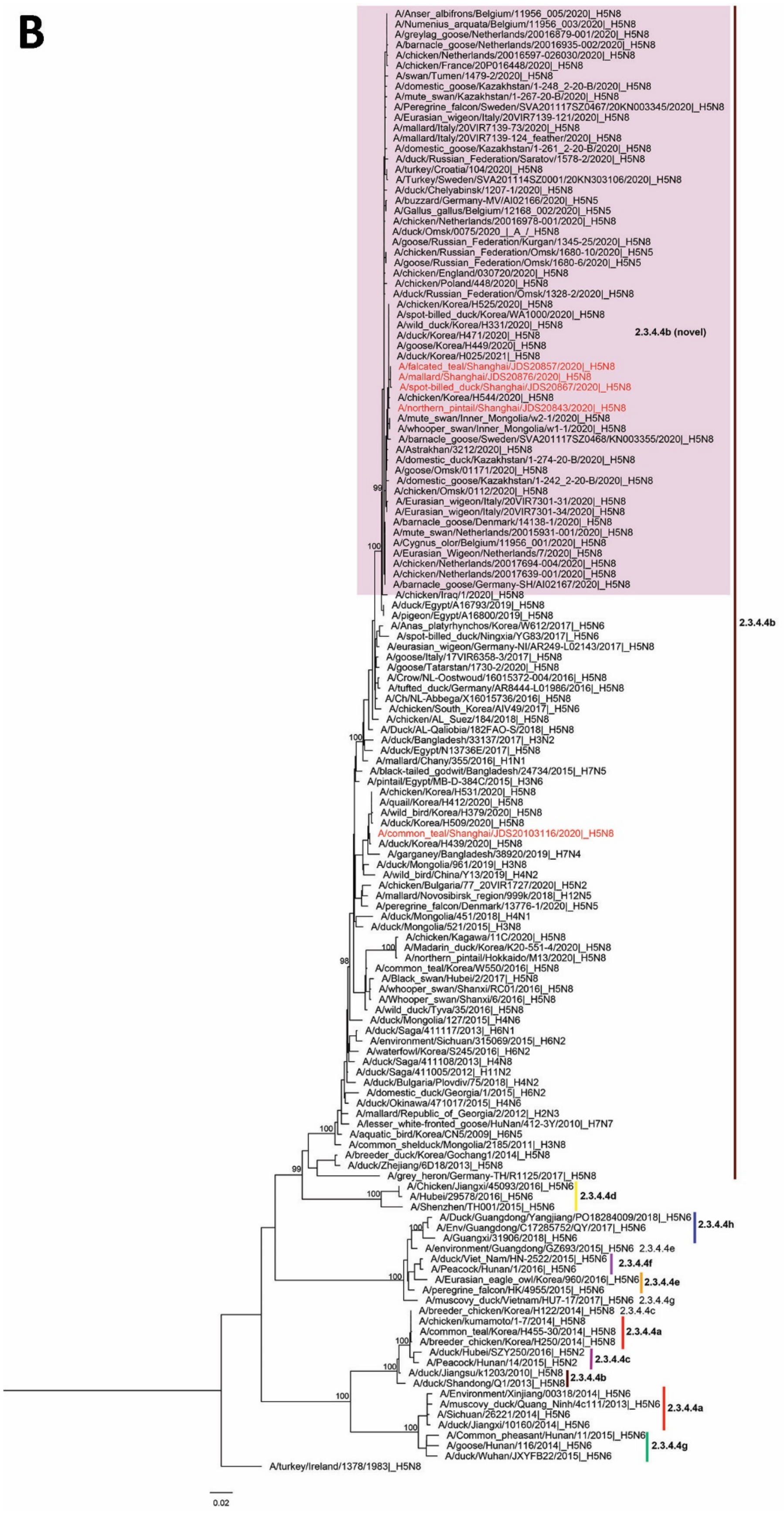
B
A/Anser_albifrons/Belgium/11956_005/2020|_H5N8
A/Numenius_arquata/Belgium/11956_003/2020|_H5N8
A/greylag_goose/Netherlands/20016879-001/2020|_H5N8
A/barnacle_goose/Netherlands/20016935-002/2020|_H5N8
A/chicken/Netherlands/20016597-026030/2020|_H5N8
A/chicken/France/20P016448/2020|_H5N8
A/swan/Tumen/1479-2/2020|_H5N8
A/domestic_goose/Kazakhstan/1-248_2-20-B/2020|_H5N8
A/mute_swan/Kazakhstan/1-267-20-B/2020|_H5N8
A/Peregrine_falcon/Sweden/SVA201117SZ0467/20KN003345/2020|_H5N8
A/Eurasian_wigeon/Italy/20VIR7139-121/2020|_H5N8
A/mallard/Italy/20VIR7139-73/2020|_H5N8
A/mallard/Italy/20VIR7139-124_feather/2020|_H5N8
A/domestic_goose/Kazakhstan/1-261_2-20-B/2020|_H5N8
A/duck/Russian_Federation/Saratov/1578-2/2020|_H5N8
A/turkey/Croatia/104/2020|_H5N8
A/Turkey/Sweden/SVA201114SZ0001/20KN303106/2020|_H5N8
A/duck/Chelyabinsk/1207-1/2020|_H5N8
A/buzzard/Germany-MV/AI02166/2020|_H5N5
A/Gallus_gallus/Belgium/12168_002/2020|_H5N5
A/chicken/Netherlands/20016978-001/2020|_H5N8
A/duck/Omsk/0075/2020_|_A_/_H5N8
A/goose/Russian_Federation/Kurgan/1345-25/2020|_H5N8
A/chicken/Russian_Federation/Omsk/1680-10/2020|_H5N5
A/goose/Russian_Federation/Omsk/1680-6/2020|_H5N5
A/chicken/England/030720/2020|_H5N8
A/chicken/Poland/448/2020|_H5N8
A/duck/Russian_Federation/Omsk/1328-2/2020|_H5N8
A/chicken/Korea/H525/2020|_H5N8
A/spot-billed_duck/Korea/WA1000/2020|_H5N8
A/wild_duck/Korea/H331/2020|_H5N8
A/duck/Korea/H471/2020|_H5N8
2.3.4.4b (novel)
A/goose/Korea/H449/2020|_H5N8
A/duck/Korea/H025/2021|_H5N8
A/falcated_teal/Shanghai/JDS20857/2020|_H5N8
A/mallard/Shanghai/JDS20876/2020|_H5N8
A/spot-billed_duck/Shanghai/JDS20867/2020|_H5N8
A/chicken/Korea/H544/2020|_H5N8
A/northern_pintail/Shanghai/JDS20843/2020|_H5N8
A/mute_swan/Inner_Mongolia/w2-1/2020|_H5N8
A/whooper_swan/Inner_Mongolia/w1-1/2020|_H5N8
A/barnacle_goose/Sweden/SVA201117SZ0468/KN003355/2020|_H5N8
A/Astrakhan/3212/2020|_H5N8
A/domestic_duck/Kazakhstan/1-274-20-B/2020|_H5N8
A/goose/Omsk/01171/2020|_H5N8
A/domestic_goose/Kazakhstan/1-242_2-20-B/2020|_H5N8
99
A/chicken/Omsk/0112/2020|_H5N8
A/Eurasian_wigeon/Italy/20VIR7301-31/2020|_H5N8
A/Eurasian_wigeon/Italy/20VIR7301-34/2020|_H5N8
A/barnacle_goose/Denmark/14138-1/2020|_H5N8
A/mute_swan/Netherlands/20015931-001/2020|_H5N8
A/Cygnus_olor/Belgium/11956_001/2020|_H5N8
100
A/Eurasian_Wigeon/Netherlands/7/2020|_H5N8
A/chicken/Netherlands/20017694-004/2020|_H5N8
A/chicken/Netherlands/20017639-001/2020|_H5N8
A/barnacle_goose/Germany-SH/AI02167/2020|_H5N8
A/chicken/Iraq/1/2020|_H5N8
A/duck/Egypt/A16793/2019|_H5N8
A/pigeon/Egypt/A16800/2019|_H5N8
A/Anas_platyrhynchos/Korea/W612/2017|_H5N6
2.3.4.4b
A/spot-billed_duck/Ningxia/YG83/2017|_H5N6
A/eurasian_wigeon/Germany-NI/AR249-L02143/2017|_H5N8
A/goose/Italy/17VIR6358-3/2017|_H5N8
A/goose/Tatarstan/1730-2/2020|_H5N8
A/Crow/NL-Oostwoud/16015372-004/2016|_H5N8
A/tufted_duck/Germany/AR8444-L01986/2016|_H5N8
A/Ch/NL-Abbega/X16015736/2016|_H5N8
A/chicken/South_Korea/AIV49/2017|_H5N6
A/chicken/AL_Suez/184/2018|_H5N8
A/Duck/AL-Qaliobia/182FAO-S/2018|_H5N8
100
A/duck/Bangladesh/33137/2017|_H3N2
A/duck/Egypt/N13736E/2017|_H5N8
A/mallard/Chany/355/2016|_H1N1
A/black-tailed_godwit/Bangladesh/24734/2015|_H7N5
A/pintail/Egypt/MB-D-384C/2015|_H3N6
A/chicken/Korea/H531/2020|_H5N8
A/quail/Korea/H412/2020|_H5N8
A/wild_bird/Korea/H379/2020|_H5N8
A/duck/Korea/H509/2020|_H5N8
A/common_teal/Shanghai/JDS20103116/2020|_H5N8
A/duck/Korea/H439/2020|_H5N8
A/garganey/Bangladesh/38920/2019|_H7N4
A/duck/Mongolia/961/2019|_H3N8
A/wild_bird/China/Y13/2019|_H4N2
A/chicken/Bulgaria/77_20VIR1727/2020|_H5N2
A/mallard/Novosibirsk_region/999k/2018|_H12N5
A/peregrine_falcon/Denmark/13776-1/2020|_H5N5
A/duck/Mongolia/451/2018|_H4N1
A/duck/Mongolia/521/2015|_H3N8
A/chicken/Kagawa/11C/2020|_H5N8
100
A/Madarin_duck/Korea/K20-551-4/2020|_H5N8
A/northern_pintail/Hokkaido/M13/2020|_H5N8
98
A/common_teal/Korea/W550/2016|_H5N8
A/Black_swan/Hubei/2/2017|_H5N8
A/whooper_swan/Shanxi/RC01/2016|_H5N8
A/Whooper_swan/Shanxi/6/2016|_H5N8
A/wild_duck/Tyva/35/2016|_H5N8
A/duck/Mongolia/127/2015|_H4N6
A/duck/Saga/411117/2013|_H6N1
A/environment/Sichuan/315069/2015|_H6N2
A/waterfowl/Korea/S245/2016|_H6N2
A/duck/Saga/411108/2013|_H4N8
A/duck/Saga/411005/2012|_H11N2
A/duck/Bulgaria/Plovdiv/75/2018|_H4N2
A/domestic_duck/Georgia/1/2015|_H6N2
A/duck/Okinawa/471017/2015|_H4N6
A/mallard/Republic_of_Georgia/2/2012|_H2N3
A/lesser_white-fronted_goose/HuNan/412-3Y/2010|_H7N7
100
A/aquatic_bird/Korea/CN5/2009|_H6N5
A/common_shelduck/Mongolia/2185/2011|_H3N8
A/breeder_duck/Korea/Gochang1/2014|_H5N8
A/duck/Zhejiang/6D18/2013|_H5N8
99
A/grey_heron/Germany-TH/R1125/2017|_H5N8
A/Chicken/Jiangxi/45093/2016|_H5N6
100
A/Hubei/29578/2016|_H5N6
2.3.4.4d
A/Shenzhen/TH001/2015|_H5N6
A/Duck/Guangdong/Yangjiang/PO18284009/2018|_H5N6
2.3.4.4h
100
A/Env/Guangdong/C17285752/QY/2017|_H5N6
A/Guangxi/31906/2018|_H5N6
A/environment/Guangdong/GZ693/2015|_H5N6 2.3.4.4e
A/duck/Viet_Nam/HN-2522/2015|_H5N6
2.3.4.4f
A/Peacock/Hunan/1/2016|_H5N6
100
A/Eurasian_eagle_owl/Korea/960/2016|_H5N6
2.3.4.4e
A/peregrine_falcon/HK/4955/2015|_H5N6
A/muscovy_duck/Vietnam/HU7-17/2017|_H5N6 2.3.4.4g
A/breeder_chicken/Korea/H122/2014|_H5N8 2.3.4.4c
A/chicken/kumamoto/1-7/2014|_H5N8
A/common_teal/Korea/H455-30/2014|_H5N8
2.3.4.4a
100
A/breeder_chicken/Korea/H250/2014|_H5N8
A/duck/Hubei/SZY250/2016|_H5N2
A/Peacock/Hunan/14/2015|_H5N2
2.3.4.4c
100
A/duck/Jiangsu/k1203/2010|_H5N8
2.3.4.4b
A/duck/Shandong/Q1/2013|_H5N8
100
A/Environment/Xinjiang/00318/2014|_H5N6
A/muscovy_duck/Quang_Ninh/4c111/2013|_H5N6
2.3.4.4a
A/Sichuan/26221/2014|_H5N6
A/duck/Jiangxi/10160/2014|_H5N6
100
A/Common_pheasant/Hunan/11/2015|_H5N6
A/goose/Hunan/116/2014|_H5N6
2.3.4.4g
A/duck/Wuhan/JXYFB22/2015|_H5N6
A/turkey/Ireland/1378/1983|_H5N8
0.02

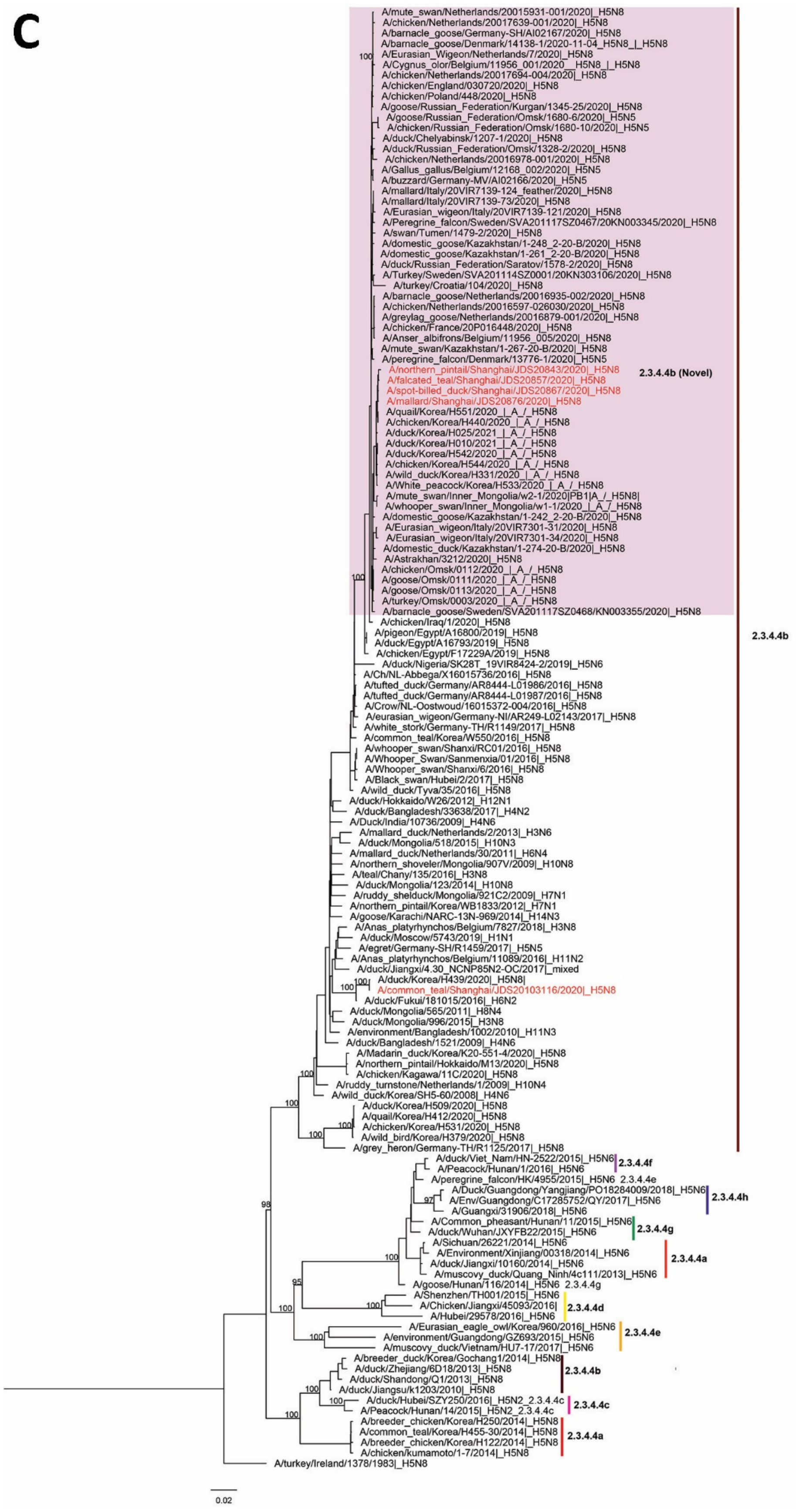
C
A/mute_swan/Netherlands/20015931-001/2020|_H5N8
A/chicken/Netherlands/20017639-001/2020|_H5N8
A/barnacle_goose/Germany-SH/AI02167/2020|_H5N8
A/barnacle_goose/Denmark/14138-1/2020-11-04_H5N8_|_H5N8
A/Eurasian_Wigeon/Netherlands/7/2020|_H5N8
A/Cygnus_olor/Belgium/11956_001/2020__H5N8_|_H5N8
A/chicken/Netherlands/20017694-004/2020|_H5N8
A/chicken/England/030720/2020|_H5N8
A/chicken/Poland/448/2020|_H5N8
A/goose/Russian_Federation/Kurgan/1345-25/2020|_H5N8
A/goose/Russian_Federation/Omsk/1680-6/2020|_H5N5
A/chicken/Russian_Federation/Omsk/1680-10/2020|_H5N5
A/duck/Chelyabinsk/1207-1/2020|_H5N8
A/duck/Russian_Federation/Omsk/1328-2/2020|_H5N8
A/chicken/Netherlands/20016978-001/2020|_H5N8
A/Gallus_gallus/Belgium/12168_002/2020|_H5N5
A/buzzard/Germany-MV/AI02166/2020|_H5N5
A/mallard/Italy/20VIR7139-124_feather/2020|_H5N8
A/mallard/Italy/20VIR7139-73/2020|_H5N8
A/Eurasian_wigeon/Italy/20VIR7139-121/2020|_H5N8
A/Peregrine_falcon/Sweden/SVA201117SZ0467/20KN003345/2020|_H5N8
A/swan/Tumen/1479-2/2020|_H5N8
A/domestic_goose/Kazakhstan/1-248_2-20-B/2020|_H5N8
A/domestic_goose/Kazakhstan/1-261_2-20-B/2020|_H5N8
A/duck/Russian_Federation/Saratov/1578-2/2020|_H5N8
A/Turkey/Sweden/SVA201114SZ0001/20KN303106/2020|_H5N8
A/turkey/Croatia/104/2020|_H5N8
A/barnacle_goose/Netherlands/20016935-002/2020|_H5N8
A/chicken/Netherlands/20016597-026030/2020|_H5N8
A/greylag_goose/Netherlands/20016879-001/2020|_H5N8
A/chicken/France/20P016448/2020|_H5N8
A/Anser_albifrons/Belgium/11956_005/2020|_H5N8
A/mute_swan/Kazakhstan/1-267-20-B/2020|_H5N8
A/peregrine_falcon/Denmark/13776-1/2020|_H5N5
A/northern_pintail/Shanghai/JDS20843/2020|_H5N8
2.3.4.4b (Novel)
A/falcated_teal/Shanghai/JDS20857/2020|_H5N8
A/spot-billed_duck/Shanghai/JDS20867/2020|_H5N8
A/mallard/Shanghai/JDS20876/2020|_H5N8
A/quail/Korea/H551/2020_|_A_/_H5N8
A/chicken/Korea/H440/2020_|_A_/_H5N8
A/duck/Korea/H025/2021_|_A_/_H5N8
A/duck/Korea/H010/2021_|_A_/_H5N8
A/duck/Korea/H542/2020_|_A_/_H5N8
A/chicken/Korea/H544/2020_|_A_/_H5N8
A/wild_duck/Korea/H331/2020_|_A_/_H5N8
A/White_peacock/Korea/H533/2020_|_A_/_H5N8
A/mute_swan/Inner_Mongolia/w2-1/2020|PB1|A_/_H5N8|
A/whooper_swan/Inner_Mongolia/w1-1/2020_|_A_/_H5N8
A/domestic_goose/Kazakhstan/1-242_2-20-B/2020|_H5N8
A/Eurasian_wigeon/Italy/20VIR7301-31/2020|_H5N8
A/Eurasian_wigeon/Italy/20VIR7301-34/2020|_H5N8
A/domestic_duck/Kazakhstan/1-274-20-B/2020|_H5N8
A/Astrakhan/3212/2020|_H5N8
A/chicken/Omsk/0112/2020_|_A_/_H5N8
A/goose/Omsk/0111/2020_|_A_/_H5N8
A/goose/Omsk/0113/2020_|_A_/_H5N8
A/turkey/Omsk/0003/2020_|_A_/_H5N8
A/barnacle_goose/Sweden/SVA201117SZ0468/KN003355/2020|_H5N8
A/chicken/Iraq/1/2020|_H5N8
A/pigeon/Egypt/A16800/2019|_H5N8
A/duck/Egypt/A16793/2019|_H5N8
A/chicken/Egypt/F17229A/2019|_H5N8
A/duck/Nigeria/SK28T_19VIR8424-2/2019|_H5N6
A/Ch/NL-Abbega/X16015736/2016|_H5N8
A/tufted_duck/Germany/AR8444-L01986/2016|_H5N8
A/tufted_duck/Germany/AR8444-L01987/2016|_H5N8
A/Crow/NL-Oostwoud/16015372-004/2016|_H5N8
A/eurasian_wigeon/Germany-NI/AR249-L02143/2017|_H5N8
A/white_stork/Germany-TH/R1149/2017|_H5N8
A/common_teal/Korea/W550/2016|_H5N8
A/whooper_swan/Shanxi/RC01/2016|_H5N8
A/Whooper_Swan/Sanmenxia/01/2016|_H5N8
A/Whooper_swan/Shanxi/6/2016|_H5N8
A/Black_swan/Hubei/2/2017|_H5N8
A/wild_duck/Tyva/35/2016|_H5N8
A/duck/Hokkaido/W26/2012|_H12N1
A/duck/Bangladesh/33638/2017|_H4N2
A/Duck/India/10736/2009|_H4N6
A/mallard_duck/Netherlands/2/2013|_H3N6
A/duck/Mongolia/518/2015|_H10N3
A/mallard_duck/Netherlands/30/2011|_H6N4
A/northern_shoveler/Mongolia/907V/2009|_H10N8
A/teal/Chany/135/2016|_H3N8
A/duck/Mongolia/123/2014|_H10N8
A/ruddy_shelduck/Mongolia/921C2/2009|_H7N1
A/northern_pintail/Korea/WB1833/2012|_H7N1
A/goose/Karachi/NARC-13N-969/2014|_H14N3
A/Anas_platyrhynchos/Belgium/7827/2018|_H3N8
A/duck/Moscow/5743/2019|_H1N1
A/egret/Germany-SH/R1459/2017|_H5N5
A/Anas_platyrhynchos/Belgium/11089/2016|_H11N2
A/duck/Jiangxi/4.30_NCNP85N2-OC/2017|_mixed
A/duck/Korea/H439/2020|_H5N8|
A/common_teal/Shanghai/JDS20103116/2020|_H5N8
A/duck/Fukui/181015/2016|_H6N2
A/duck/Mongolia/565/2011|_H8N4
A/duck/Mongolia/996/2015|_H3N8
A/environment/Bangladesh/1002/2010|_H11N3
A/duck/Bangladesh/1521/2009|_H4N6
A/Madarin_duck/Korea/K20-551-4/2020|_H5N8
A/northern_pintail/Hokkaido/M13/2020|_H5N8
A/chicken/Kagawa/11C/2020|_H5N8
A/ruddy_turnstone/Netherlands/1/2009|_H10N4
A/wild_duck/Korea/SH5-60/2008|_H4N6
A/duck/Korea/H509/2020|_H5N8
A/quail/Korea/H412/2020|_H5N8
A/chicken/Korea/H531/2020|_H5N8
A/wild_bird/Korea/H379/2020|_H5N8
A/grey_heron/Germany-TH/R1125/2017|_H5N8
2.3.4.4b
A/duck/Viet_Nam/HN-2522/2015|_H5N6
A/Peacock/Hunan/1/2016|_H5N6
2.3.4.4f
A/peregrine_falcon/HK/4955/2015|_H5N6 2.3.4.4e
A/Duck/Guangdong/Yangjiang/PO18284009/2018|_H5N6
A/Env/Guangdong/C17285752/QY/2017|_H5N6
A/Guangxi/31906/2018|_H5N6
2.3.4.4h
A/Common_pheasant/Hunan/11/2015|_H5N6
A/duck/Wuhan/JXYFB22/2015|_H5N6
2.3.4.4g
A/Sichuan/26221/2014|_H5N6
A/Environment/Xinjiang/00318/2014|_H5N6
A/duck/Jiangxi/10160/2014|_H5N6
A/muscovy_duck/Quang_Ninh/4c111/2013|_H5N6
2.3.4.4a
A/goose/Hunan/116/2014|_H5N6 2.3.4.4g
A/Shenzhen/TH001/2015|_H5N6
A/Chicken/Jiangxi/45093/2016|
A/Hubei/29578/2016|_H5N6
2.3.4.4d
A/Eurasian_eagle_owl/Korea/960/2016|_H5N6
A/environment/Guangdong/GZ693/2015|_H5N6
A/muscovy_duck/Vietnam/HU7-17/2017|_H5N6
2.3.4.4e
A/breeder_duck/Korea/Gochang1/2014|_H5N8
A/duck/Zhejiang/6D18/2013|_H5N8
A/duck/Shandong/Q1/2013|_H5N8
A/duck/Jiangsu/k1203/2010|_H5N8
2.3.4.4b
A/duck/Hubei/SZY250/2016|_H5N2_2.3.4.4c
A/Peacock/Hunan/14/2015|_H5N2_2.3.4.4c
2.3.4.4c
A/breeder_chicken/Korea/H250/2014|_H5N8
A/common_teal/Korea/H455-30/2014|_H5N8
A/breeder_chicken/Korea/H122/2014|_H5N8
A/chicken/kumamoto/1-7/2014|_H5N8
2.3.4.4a
A/turkey/Ireland/1378/1983|_H5N8
100
98
95
97
0.02

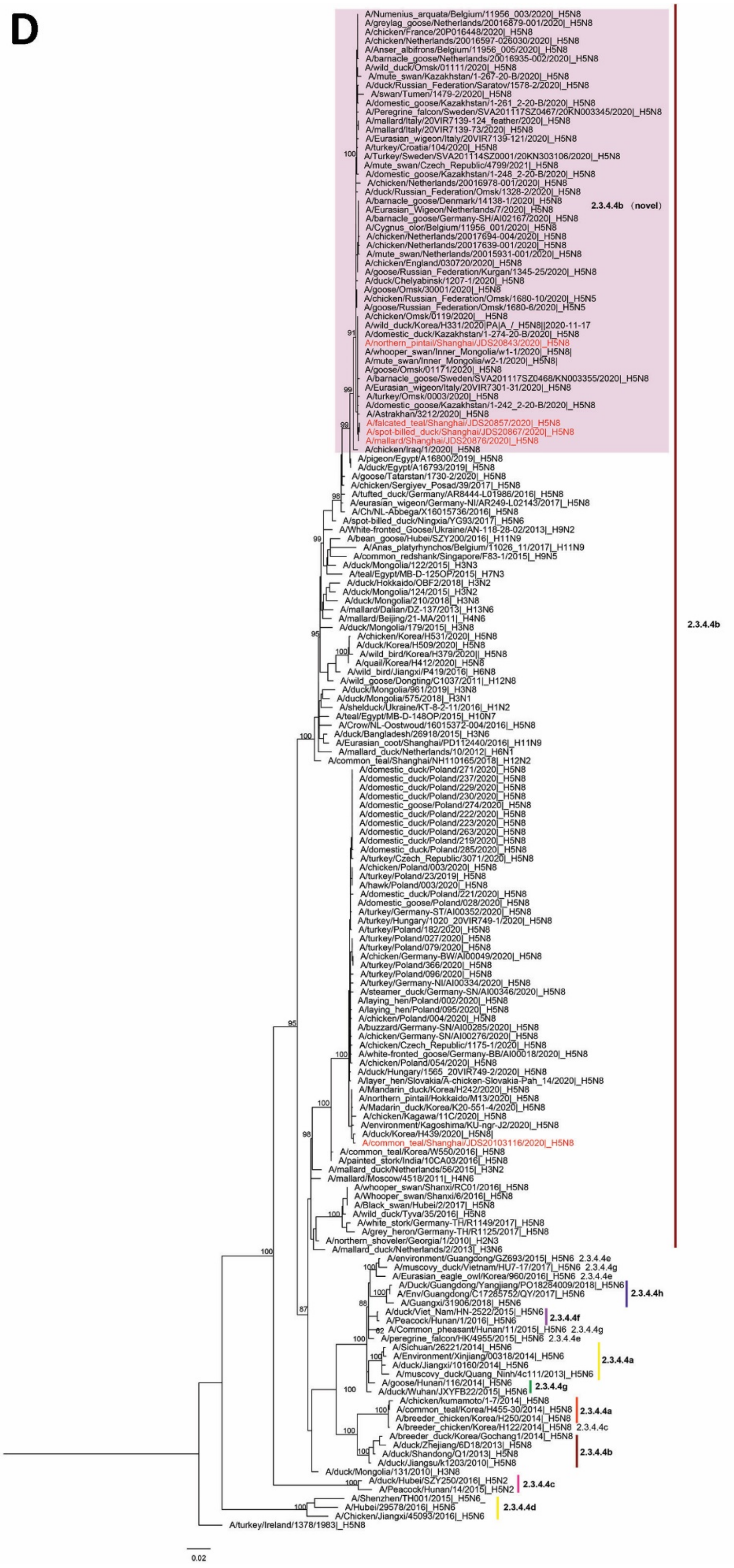
D
A/Numenius_arquata/Belgium/11956_003/2020|_H5N8
A/greylag_goose/Netherlands/20016879-001/2020|_H5N8
A/chicken/France/20P016448/2020|_H5N8
A/chicken/Netherlands/20016597-026030/2020|_H5N8
A/Anser_albifrons/Belgium/11956_005/2020|_H5N8
A/barnacle_goose/Netherlands/20016935-002/2020|_H5N8
A/wild_duck/Omsk/01111/2020|_H5N8
A/mute_swan/Kazakhstan/1-267-20-B/2020|_H5N8
A/duck/Russian_Federation/Saratov/1578-2/2020|_H5N8
A/swan/Tumen/1479-2/2020|_H5N8
A/domestic_goose/Kazakhstan/1-261_2-20-B/2020|_H5N8
A/Peregrine_falcon/Sweden/SVA201117SZ0467/20KN003345/2020|_H5N8
A/mallard/Italy/20VIR7139-124_feather/2020|_H5N8
A/mallard/Italy/20VIR7139-73/2020|_H5N8
A/Eurasian_wigeon/Italy/20VIR7139-121/2020|_H5N8
A/turkey/Croatia/104/2020|_H5N8
100
A/Turkey/Sweden/SVA201114SZ0001/20KN303106/2020|_H5N8
A/mute_swan/Czech_Republic/4799/2021|_H5N8
A/domestic_goose/Kazakhstan/1-248_2-20-B/2020|_H5N8
A/chicken/Netherlands/20016978-001/2020|_H5N8
A/duck/Russian_Federation/Omsk/1328-2/2020|_H5N8
A/barnacle_goose/Denmark/14138-1/2020|_H5N8
A/Eurasian_Wigeon/Netherlands/7/2020|_H5N8
2.3.4.4b (novel)
A/barnacle_goose/Germany-SH/AI02167/2020|_H5N8
A/Cygnus_olor/Belgium/11956_001/2020|_H5N8
A/chicken/Netherlands/20017694-004/2020|_H5N8
A/chicken/Netherlands/20017639-001/2020|_H5N8
A/mute_swan/Netherlands/20015931-001/2020|_H5N8
A/chicken/England/030720/2020|_H5N8
A/goose/Russian_Federation/Kurgan/1345-25/2020|_H5N8
A/duck/Chelyabinsk/1207-1/2020|_H5N8
A/goose/Omsk/30001/2020|_H5N8
A/chicken/Russian_Federation/Omsk/1680-10/2020|_H5N5
A/goose/Russian_Federation/Omsk/1680-6/2020|_H5N5
A/chicken/Omsk/0119/2020|__H5N8
A/wild_duck/Korea/H331/2020|PA|A_/_H5N8||2020-11-17
91
A/domestic_duck/Kazakhstan/1-274-20-B/2020|_H5N8
A/northern_pintail/Shanghai/JDS20843/2020|_H5N8
A/whooper_swan/Inner_Mongolia/w1-1/2020|_H5N8|
A/mute_swan/Inner_Mongolia/w2-1/2020|_H5N8|
A/goose/Omsk/01171/2020|_H5N8
A/barnacle_goose/Sweden/SVA201117SZ0468/KN003355/2020|_H5N8
99
A/Eurasian_wigeon/Italy/20VIR7301-31/2020|_H5N8
A/turkey/Omsk/0003/2020|_H5N8
A/domestic_goose/Kazakhstan/1-242_2-20-B/2020|_H5N8
A/Astrakhan/3212/2020|_H5N8
99
A/falcated_teal/Shanghai/JDS20857/2020|_H5N8
A/spot-billed_duck/Shanghai/JDS20867/2020|_H5N8
A/mallard/Shanghai/JDS20876/2020|_H5N8
A/chicken/Iraq/1/2020|_H5N8
A/pigeon/Egypt/A16800/2019|_H5N8
A/duck/Egypt/A16793/2019|_H5N8
A/goose/Tatarstan/1730-2/2020|_H5N8
A/chicken/Sergiyev_Posad/39/2017|_H5N8
98
A/tufted_duck/Germany/AR8444-L01986/2016|_H5N8
A/eurasian_wigeon/Germany-NI/AR249-L02143/2017|_H5N8
A/Ch/NL-Abbega/X16015736/2016|_H5N8
A/spot-billed_duck/Ningxia/YG93/2017|_H5N6
A/White-fronted_Goose/Ukraine/AN-118-28-02/2013|_H9N2
99
A/bean_goose/Hubei/SZY200/2016|_H11N9
A/Anas_platyrhynchos/Belgium/11026_11/2017|_H11N9
A/common_redshank/Singapore/F83-1/2015|_H9N5
A/duck/Mongolia/122/2015|_H3N3
A/teal/Egypt/MB-D-125OP/2015|_H7N3
A/duck/Hokkaido/OBF2/2018|_H3N2
A/duck/Mongolia/124/2015|_H3N2
A/duck/Mongolia/210/2018|_H3N8
A/mallard/Dalian/DZ-137/2013|_H13N6
A/mallard/Beijing/21-MA/2011|_H4N6
A/duck/Mongolia/179/2015|_H3N8
95
A/chicken/Korea/H531/2020|_H5N8
A/duck/Korea/H509/2020|_H5N8
100
A/wild_bird/Korea/H379/2020||_H5N8
A/quail/Korea/H412/2020|_H5N8
A/wild_bird/Jiangxi/P419/2016|_H6N8
A/wild_goose/Dongting/C1037/2011|_H12N8
A/duck/Mongolia/961/2019|_H3N8
A/duck/Mongolia/575/2018|_H3N1
A/shelduck/Ukraine/KT-8-2-11/2016|_H1N2
A/teal/Egypt/MB-D-148OP/2015|_H10N7
A/Crow/NL-Oostwoud/16015372-004/2016|_H5N8
A/duck/Bangladesh/26918/2015|_H3N6
100
A/Eurasian_coot/Shanghai/PD1T2440/2016|_H11N9
A/mallard_duck/Netherlands/10/2012|_H6N1
A/common_teal/Shanghai/NH110165/2018|_H12N2
2.3.4.4b
A/domestic_duck/Poland/271/2020|_H5N8
A/domestic_duck/Poland/237/2020|_H5N8
A/domestic_duck/Poland/229/2020|_H5N8
A/domestic_duck/Poland/230/2020|_H5N8
A/domestic_goose/Poland/274/2020|_H5N8
A/domestic_duck/Poland/222/2020|_H5N8
A/domestic_duck/Poland/223/2020|_H5N8
A/domestic_duck/Poland/263/2020|_H5N8
A/domestic_duck/Poland/219/2020|_H5N8
A/domestic_duck/Poland/285/2020|_H5N8
A/turkey/Czech_Republic/3071/2020|_H5N8
A/chicken/Poland/003/2020|_H5N8
A/turkey/Poland/23/2019|_H5N8
A/hawk/Poland/003/2020|_H5N8
A/domestic_duck/Poland/221/2020|_H5N8
A/domestic_goose/Poland/028/2020|_H5N8
A/turkey/Germany-ST/AI00352/2020|_H5N8
A/turkey/Hungary/1020_20VIR749-1/2020|_H5N8
A/turkey/Poland/182/2020|_H5N8
A/turkey/Poland/027/2020|_H5N8
A/turkey/Poland/079/2020|_H5N8
A/chicken/Germany-BW/AI00049/2020|_H5N8
A/turkey/Poland/366/2020|_H5N8
A/turkey/Poland/096/2020|_H5N8
A/turkey/Germany-NI/AI00334/2020|_H5N8
A/steamer_duck/Germany-SN/AI00346/2020|_H5N8
A/laying_hen/Poland/002/2020|_H5N8
A/laying_hen/Poland/095/2020|_H5N8
95
A/chicken/Poland/004/2020|_H5N8
A/buzzard/Germany-SN/AI00285/2020|_H5N8
A/chicken/Germany-SN/AI00276/2020|_H5N8
A/chicken/Czech_Republic/1175-1/2020|_H5N8
100
A/white-fronted_goose/Germany-BB/AI00018/2020|_H5N8
A/chicken/Poland/054/2020|_H5N8
A/duck/Hungary/1565_20VIR749-2/2020|_H5N8
A/layer_hen/Slovakia/A-chicken-Slovakia-Pah_14/2020|_H5N8
A/Mandarin_duck/Korea/H242/2020|_H5N8
A/northern_pintail/Hokkaido/M13/2020|_H5N8
100
A/Madarin_duck/Korea/K20-551-4/2020|_H5N8
A/chicken/Kagawa/11C/2020|_H5N8
A/environment/Kagoshima/KU-ngr-J2/2020|_H5N8
98
A/duck/Korea/H439/2020|_H5N8|
A/common_teal/Shanghai/JDS20103116/2020|_H5N8
A/common_teal/Korea/W550/2016|_H5N8
A/painted_stork/India/10CA03/2016|_H5N8
A/mallard_duck/Netherlands/56/2015|_H3N2
A/mallard/Moscow/4518/2011|_H4N6
A/whooper_swan/Shanxi/RC01/2016|_H5N8
A/Whooper_swan/Shanxi/6/2016|_H5N8
A/Black_swan/Hubei/2/2017|_H5N8
100
A/wild_duck/Tyva/35/2016|_H5N8
A/white_stork/Germany-TH/R1149/2017|_H5N8
A/grey_heron/Germany-TH/R1125/2017|_H5N8
A/northern_shoveler/Georgia/1/2010|_H2N3
100
A/mallard_duck/Netherlands/2/2013|_H3N6
A/environment/Guangdong/GZ693/2015|_H5N6 2.3.4.4e
100
A/muscovy_duck/Vietnam/HU7-17/2017|_H5N6 2.3.4.4g
A/Eurasian_eagle_owl/Korea/960/2016|_H5N6 2.3.4.4e
A/Duck/Guangdong/Yangjiang/PO18284009/2018|_H5N6
100
A/Env/Guangdong/C17285752/QY/2017|_H5N6
2.3.4.4h
A/Guangxi/31906/2018|_H5N6
87
88
A/duck/Viet_Nam/HN-2522/2015|_H5N6
2.3.4.4f
A/Peacock/Hunan/1/2016|_H5N6
62
A/Common_pheasant/Hunan/11/2015|_H5N6 2.3.4.4g
100
A/peregrine_falcon/HK/4955/2015|_H5N6 2.3.4.4e
A/Sichuan/26221/2014|_H5N6
100
A/Environment/Xinjiang/00318/2014|_H5N6
2.3.4.4a
A/duck/Jiangxi/10160/2014|_H5N6
A/muscovy_duck/Quang_Ninh/4c111/2013|_H5N6
100
A/goose/Hunan/116/2014|_H5N6
2.3.4.4g
A/duck/Wuhan/JXYFB22/2015|_H5N6
A/chicken/kumamoto/1-7/2014|_H5N8
100
A/common_teal/Korea/H455-30/2014|_H5N8
2.3.4.4a
A/breeder_chicken/Korea/H250/2014|_H5N8
A/breeder_chicken/Korea/H122/2014|_H5N8 2.3.4.4c
100
A/breeder_duck/Korea/Gochang1/2014|_H5N8
A/duck/Zhejiang/6D18/2013|_H5N8
100
A/duck/Shandong/Q1/2013|_H5N8
2.3.4.4b
A/duck/Jiangsu/k1203/2010|_H5N8
A/duck/Mongolia/131/2010|_H3N8
100
A/duck/Hubei/SZY250/2016|_H5N2
2.3.4.4c
A/Peacock/Hunan/14/2015|_H5N2
A/Shenzhen/TH001/2015|_H5N6
100
A/Hubei/29578/2016|_H5N6
2.3.4.4d
A/Chicken/Jiangxi/45093/2016|_H5N6
A/turkey/Ireland/1378/1983|_H5N8
0.02

E

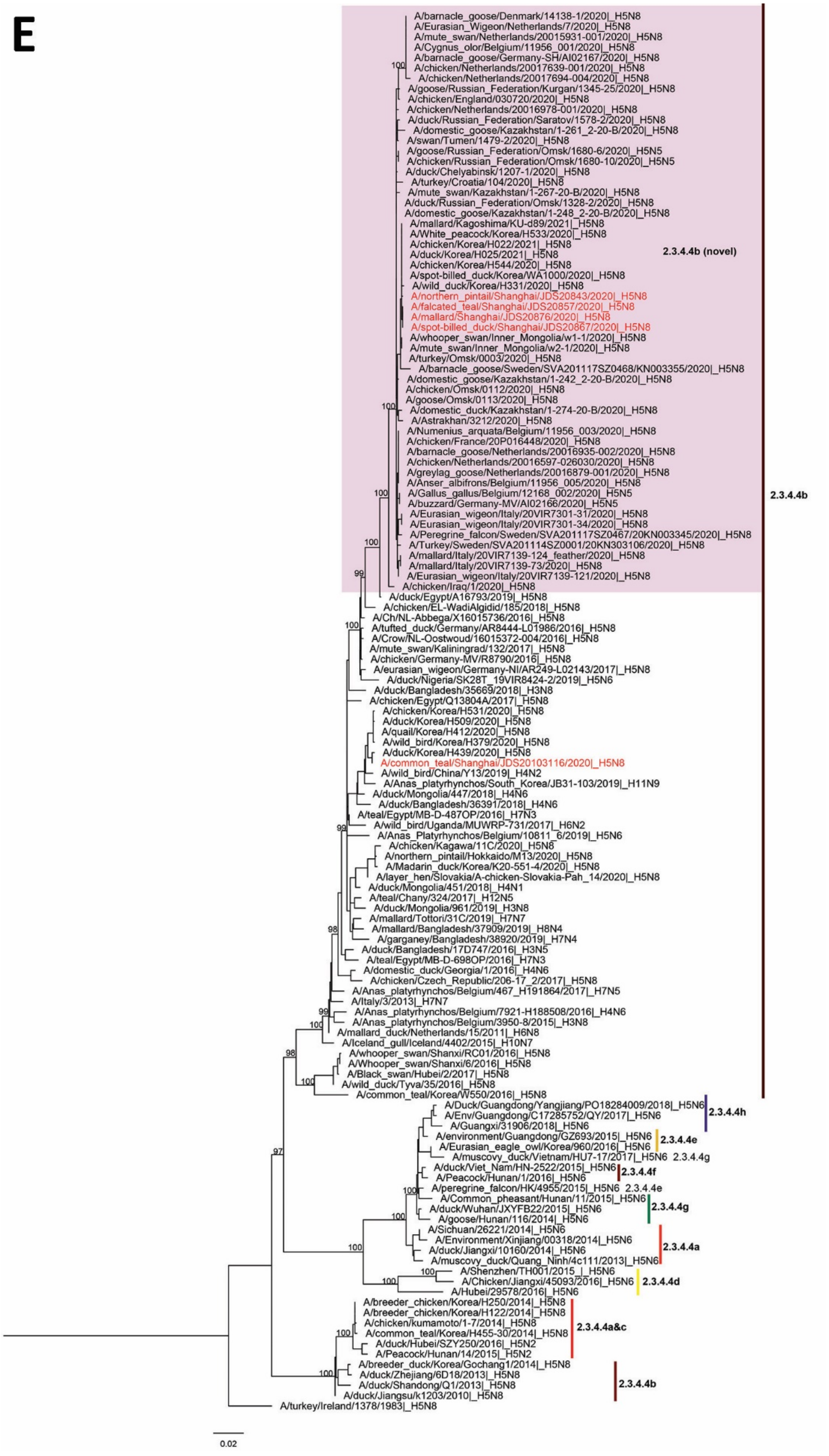
A/barnacle_goose/Denmark/14138-1/2020|_H5N8
A/Eurasian_Wigeon/Netherlands/7/2020|_H5N8
A/mute_swan/Netherlands/20015931-001/2020|_H5N8
A/Cygnus_olor/Belgium/11956_001/2020|_H5N8
A/barnacle_goose/Germany-SH/AI02167/2020|_H5N8
A/chicken/Netherlands/20017639-001/2020|_H5N8
A/chicken/Netherlands/20017694-004/2020|_H5N8
A/goose/Russian_Federation/Kurgan/1345-25/2020|_H5N8
A/chicken/England/030720/2020|_H5N8
A/chicken/Netherlands/20016978-001/2020|_H5N8
A/duck/Russian_Federation/Saratov/1578-2/2020|_H5N8
A/domestic_goose/Kazakhstan/1-261_2-20-B/2020|_H5N8
A/swan/Tumen/1479-2/2020|_H5N8
A/goose/Russian_Federation/Omsk/1680-6/2020|_H5N5
A/chicken/Russian_Federation/Omsk/1680-10/2020|_H5N5
A/duck/Chelyabinsk/1207-1/2020|_H5N8
A/turkey/Croatia/104/2020|_H5N8
A/mute_swan/Kazakhstan/1-267-20-B/2020|_H5N8
A/duck/Russian_Federation/Omsk/1328-2/2020|_H5N8
A/domestic_goose/Kazakhstan/1-248_2-20-B/2020|_H5N8
A/mallard/Kagoshima/KU-d89/2021|_H5N8
A/White_peacock/Korea/H533/2020|_H5N8
A/chicken/Korea/H022/2021|_H5N8
A/duck/Korea/H025/2021|_H5N8
A/chicken/Korea/H544/2020|_H5N8
A/spot-billed_duck/Korea/WA1000/2020|_H5N8
A/wild_duck/Korea/H331/2020|_H5N8
A/northern_pintail/Shanghai/JDS20843/2020|_H5N8
A/falcated_teal/Shanghai/JDS20857/2020|_H5N8
A/mallard/Shanghai/JDS20876/2020|_H5N8
A/spot-billed_duck/Shanghai/JDS20867/2020|_H5N8
A/whooper_swan/Inner_Mongolia/w1-1/2020|_H5N8
A/mute_swan/Inner_Mongolia/w2-1/2020|_H5N8
A/turkey/Omsk/0003/2020|_H5N8
A/barnacle_goose/Sweden/SVA201117SZ0468/KN003355/2020|_H5N8
A/domestic_goose/Kazakhstan/1-242_2-20-B/2020|_H5N8
A/chicken/Omsk/0112/2020|_H5N8
A/goose/Omsk/0113/2020|_H5N8
A/domestic_duck/Kazakhstan/1-274-20-B/2020|_H5N8
A/Astrakhan/3212/2020|_H5N8
A/Numenius_arquata/Belgium/11956_003/2020|_H5N8
A/chicken/France/20P016448/2020|_H5N8
A/barnacle_goose/Netherlands/20016935-002/2020|_H5N8
A/chicken/Netherlands/20016597-026030/2020|_H5N8
A/greylag_goose/Netherlands/20016879-001/2020|_H5N8
A/Anser_albifrons/Belgium/11956_005/2020|_H5N8
A/Gallus_gallus/Belgium/12168_002/2020|_H5N8
A/buzzard/Germany-MV/AI02166/2020|_H5N5
A/Eurasian_wigeon/Italy/20VIR7301-31/2020|_H5N8
A/Eurasian_wigeon/Italy/20VIR7301-34/2020|_H5N8
A/Peregrine_falcon/Sweden/SVA201117SZ0467/20KN003345/2020|_H5N8
A/Turkey/Sweden/SVA201114SZ0001/20KN303106/2020|_H5N8
A/mallard/Italy/20VIR7139-124_feather/2020|_H5N8
A/mallard/Italy/20VIR7139-73/2020|_H5N8
A/Eurasian_wigeon/Italy/20VIR7139-121/2020|_H5N8
A/chicken/Iraq/1/2020|_H5N8
2.3.4.4b (novel)
A/duck/Egypt/A16793/2019|_H5N8
A/chicken/EL-WadiAlgidid/185/2018|_H5N8
A/Ch/NL-Abbega/X16015736/2016|_H5N8
A/tufted_duck/Germany/AR8444-L01986/2016|_H5N8
A/Crow/NL-Oostwoud/16015372-004/2016|_H5N8
A/mute_swan/Kaliningrad/132/2017|_H5N8
A/chicken/Germany-MV/R8790/2016|_H5N8
A/eurasian_wigeon/Germany-NI/AR249-L02143/2017|_H5N8
A/duck/Nigeria/SK28T_19VIR8424-2/2019|_H5N6
A/duck/Bangladesh/35669/2018|_H3N8
A/chicken/Egypt/Q13804A/2017|_H5N8
A/chicken/Korea/H531/2020|_H5N8
A/duck/Korea/H509/2020|_H5N8
A/quail/Korea/H412/2020|_H5N8
A/wild_bird/Korea/H379/2020|_H5N8
A/duck/Korea/H439/2020|_H5N8
A/common_teal/Shanghai/JDS20103116/2020|_H5N8
A/wild_bird/China/Y13/2019|_H4N2
A/Anas_platyrhynchos/South_Korea/JB31-103/2019|_H11N9
A/duck/Mongolia/447/2018|_H4N6
A/duck/Bangladesh/36391/2018|_H4N6
A/teal/Egypt/MB-D-487OP/2016|_H7N3
A/wild_bird/Uganda/MUWRP-731/2017|_H6N2
A/Anas_Platyrhynchos/Belgium/10811_6/2019|_H5N6
A/chicken/Kagawa/11C/2020|_H5N8
A/northern_pintail/Hokkaido/M13/2020|_H5N8
A/Madarin_duck/Korea/K20-551-4/2020|_H5N8
A/layer_hen/Slovakia/A-chicken-Slovakia-Pah_14/2020|_H5N8
A/duck/Mongolia/451/2018|_H4N1
A/teal/Chany/324/2017|_H12N5
A/duck/Mongolia/961/2019|_H3N8
A/mallard/Tottori/31C/2019|_H7N7
A/mallard/Bangladesh/37909/2019|_H8N4
A/garganey/Bangladesh/38920/2019|_H7N4
A/duck/Bangladesh/17D747/2016|_H3N5
A/teal/Egypt/MB-D-698OP/2016|_H7N3
A/domestic_duck/Georgia/1/2016|_H4N6
A/chicken/Czech_Republic/206-17_2/2017|_H5N8
A/Anas_platyrhynchos/Belgium/467_H191864/2017|_H7N5
A/Italy/3/2013|_H7N7
A/Anas_platyrhynchos/Belgium/7921-H188508/2016|_H4N6
A/Anas_platyrhynchos/Belgium/3950-8/2015|_H3N8
A/mallard_duck/Netherlands/15/2011|_H6N8
A/Iceland_gull/Iceland/4402/2015|_H10N7
A/whooper_swan/Shanxi/RC01/2016|_H5N8
A/Whooper_swan/Shanxi/6/2016|_H5N8
A/Black_swan/Hubei/2/2017|_H5N8
A/wild_duck/Tyva/35/2016|_H5N8
A/common_teal/Korea/W550/2016|_H5N8
2.3.4.4b
A/Duck/Guangdong/Yangjiang/PO18284009/2018|_H5N6
A/Env/Guangdong/C17285752/QY/2017|_H5N6
A/Guangxi/31906/2018|_H5N6
2.3.4.4h
A/environment/Guangdong/GZ693/2015|_H5N6
A/Eurasian_eagle_owl/Korea/960/2016|_H5N6
2.3.4.4e
A/muscovy_duck/Vietnam/HU7-17/2017|_H5N6 2.3.4.4g
A/duck/Viet_Nam/HN-2522/2015|_H5N6
A/Peacock/Hunan/1/2016|_H5N6
2.3.4.4f
A/peregrine_falcon/HK/4955/2015|_H5N6 2.3.4.4e
A/Common_pheasant/Hunan/11/2015|_H5N6
A/duck/Wuhan/JXYFB22/2015|_H5N6
A/goose/Hunan/116/2014|_H5N6
2.3.4.4g
A/Sichuan/26221/2014|_H5N6
A/Environment/Xinjiang/00318/2014|_H5N6
A/duck/Jiangxi/10160/2014|_H5N6
A/muscovy_duck/Quang_Ninh/4c111/2013|_H5N6
2.3.4.4a
A/Shenzhen/TH001/2015_|_H5N6
A/Chicken/Jiangxi/45093/2016|_H5N6
A/Hubei/29578/2016|_H5N6
2.3.4.4d
A/breeder_chicken/Korea/H250/2014|_H5N8
A/breeder_chicken/Korea/H122/2014|_H5N8
A/chicken/kumamoto/1-7/2014|_H5N8
A/common_teal/Korea/H455-30/2014|_H5N8
A/duck/Hubei/SZY250/2016|_H5N2
A/Peacock/Hunan/14/2015|_H5N2
2.3.4.4a&c
A/breeder_duck/Korea/Gochang1/2014|_H5N8
A/duck/Zhejiang/6D18/2013|_H5N8
A/duck/Shandong/Q1/2013|_H5N8
A/duck/Jiangsu/k1203/2010|_H5N8
2.3.4.4b
A/turkey/Ireland/1378/1983|_H5N8
0.02

F

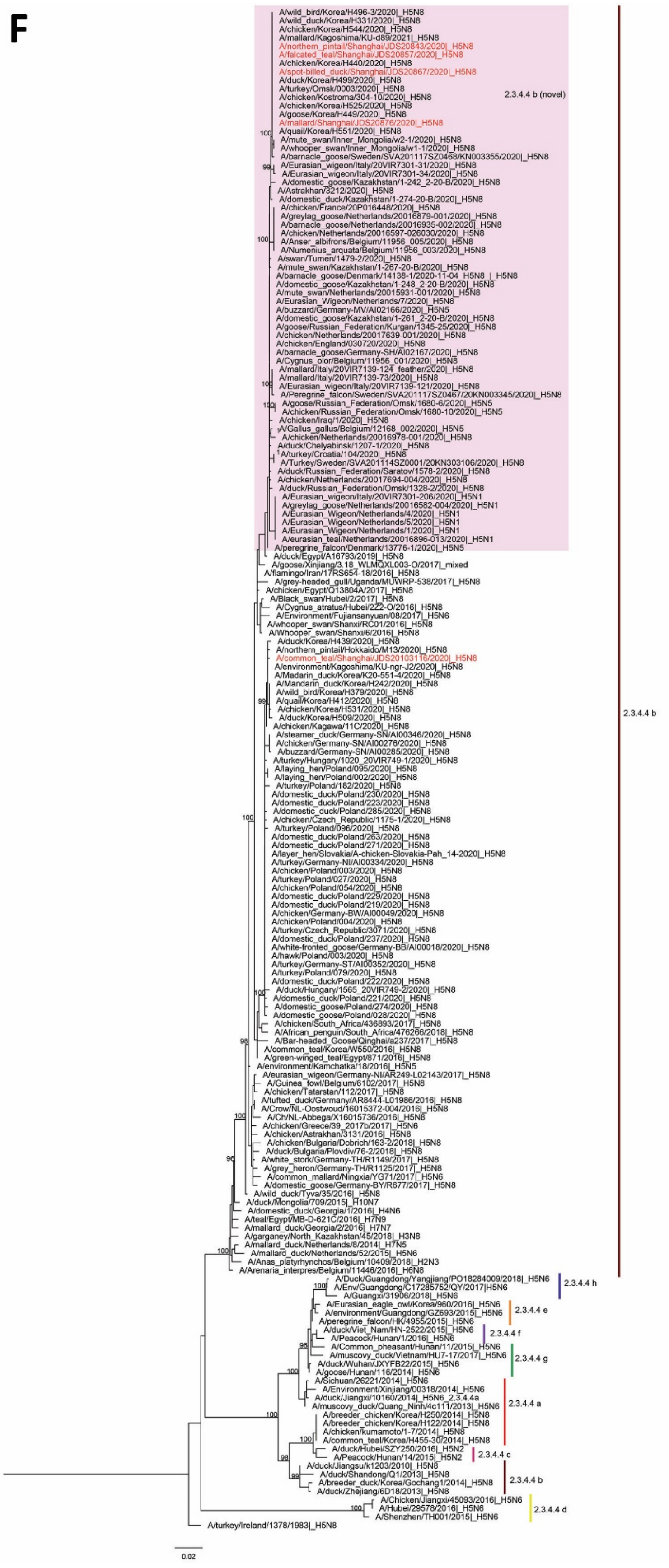
2.3.4.4 b (novel)
2.3.4.4 b
2.3.4.4 h
2.3.4.4 e
2.3.4.4 f
2.3.4.4 g
2.3.4.4 a
2.3.4.4 c
2.3.4.4 b
2.3.4.4 d
0.02

G

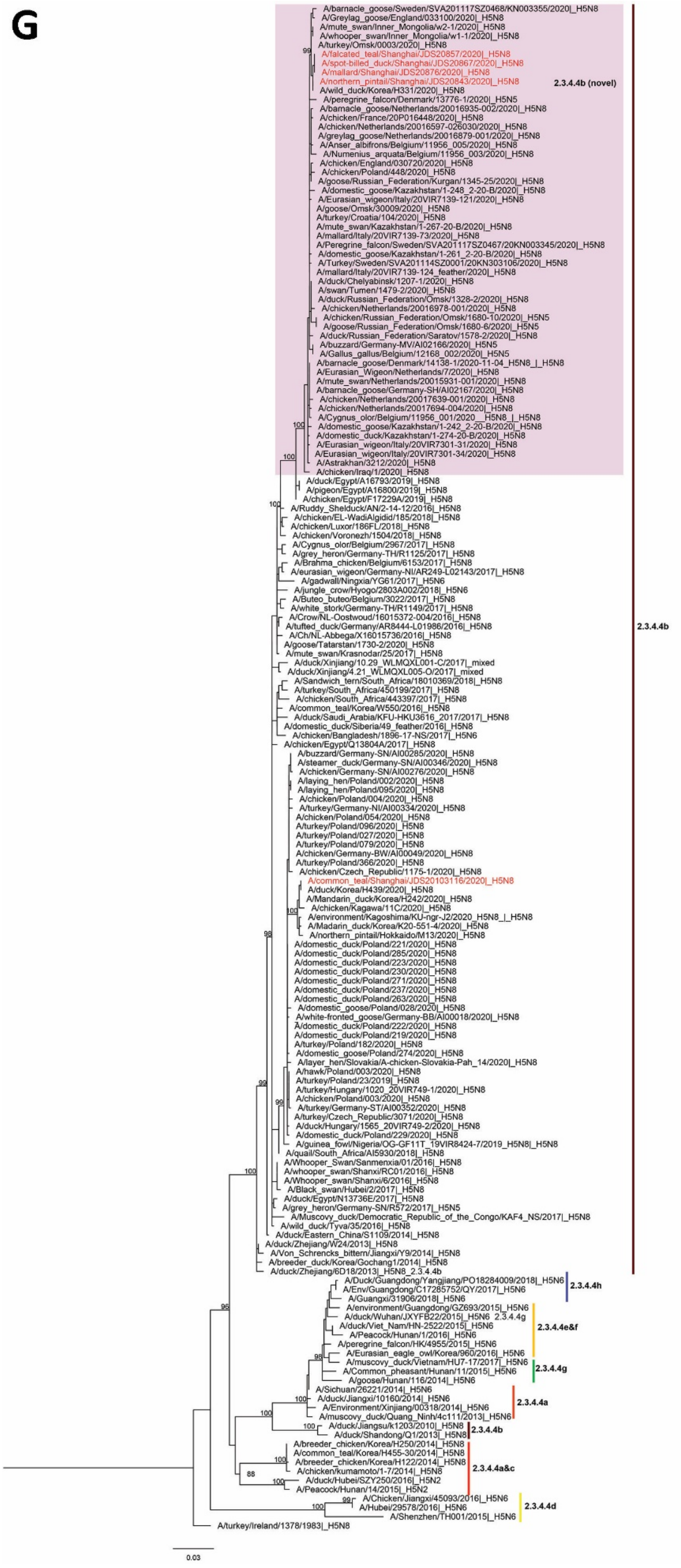
2.3.4.4b (novel)
2.3.4.4b
2.3.4.4h
2.3.4.4e&f
2.3.4.4g
2.3.4.4a
2.3.4.4b
2.3.4.4a&c
2.3.4.4d
0.03

Appendix Figure. Phylogenetic trees of highly pathogenic avian influenza A(H5N8) isolated in Shanghai, China during 2020 (red text), and reference sequences in GISAID (https://www.gisaid.org). Tree was based on 7 gene segments; it was constructed using IQ-TREE (*1*) with 1,000 bootstrap replicates. Labels indicate subclades 2.3.4.4a–h. Pink shading indicates novel genotypes of clade 2.3.4.4b. Numbers to the left of node indicate bootstrap values. Scale bars indicate average number of nucleotide substitutions per site.

Reference

1. Minh BQ, Schmidt HA, Chernomor O, Schrempf D, Woodhams MD, von Haeseler A, et al. IQ-TREE 2: new models and efficient methods for phylogenetic inference in the genomic era. Mol Biol Evol. 2020;37:1530–4. PubMed https://doi.org/10.1093/molbev/msaa015
